# Incidence and Impact of Musculoskeletal Complaints in Type 2 Diabetic Australian Aboriginal Populations

**DOI:** 10.7759/cureus.91055

**Published:** 2025-08-26

**Authors:** Aiman A Ishaq, Syed M Ahsan, Neil Ashwood

**Affiliations:** 1 Gastroenterology, University Hospitals of Derby and Burton National Health Service Foundation Trust, Derby, GBR; 2 General Practice, Uralla Clinic, Uralla, AUS; 3 General Practice, Armajun Aboriginal Health Service, Armidale, AUS; 4 Trauma and Orthopaedics, University Hospitals of Derby and Burton National Health Service Foundation Trust, Derby, GBR

**Keywords:** aboriginal community, diabetes type 2, musculoskeletal disorder, musculoskeletal problems, pain perception, type 2 diabetes

## Abstract

Background

Aboriginal populations are those indigenous to Australia and are at greater risk of having diabetes. Diabetes is known to cause and contribute to numerous musculoskeletal (MSK) complaints. Data on such populations are scarce, and MSK pathologies and diabetes mellitus can both have a significant impact on mortality and morbidity.

Aims

The primary aim was to assess the prevalence and severity of diabetes mellitus in Aboriginal populations and the number and type of MSK complaints experienced by such patients. Secondary aims included looking at the pain threshold of such patients and their correlation with the severity of their diabetes. The smoking status of patients was also recorded.

Methods

This study was conducted as a retrospective cross-sectional study. This was performed using the patient information software system available at the Armajun trust called Communicare. The software contained patient records, including imaging, and confirmed diagnoses. A modified pain scoring system was used to score pain based on the World Health Organization (WHO) pain ladder, using clinician-acquired data. The number and type of MSK complaints were recorded. Individual results were also recorded for patients in each centre. A total of 222 patients were identified in the initial sample, of which 167 met the inclusion criteria (75%).

Results

Most patients had poor/ adequate HbA1c control. And pain scores were most commonly low. However, patients with poor control did have higher scores on average across most centres. In MSK complaints, chronic lower back pain, osteoarthritis and gout were the most common.

Conclusion

Fundamentally, we observed that the severity of diabetes does not have a significant impact on MSK complaint quantity and quality. With regards to the secondary aim, pain does not seem to be correlated with the degree of glycaemic control or the insulin dependence of a diabetic Aboriginal patient.

## Introduction

Diabetes is one of the most common chronic diseases, reaching worldwide pandemic status, and is associated with several complications [1-5]. Diabetic complications can be subdivided in many ways, one of which is macrovascular and microvascular. From the vast list of microvascular complications of diabetes, there exist conditions such as diabetic neuropathy and diabetic foot [6]. Diabetes is the leading cause of musculoskeletal (MSK) complaints, from wound and fracture healing to Charcot arthropathy. MSK complaints are the leading contributor to disability worldwide, especially in the developed world [7]. Moreover, due to recent demographic shifts, the incidence and prevalence of MSK diseases are only increasing, significantly impacting patients' quality of life.

The link between diabetes and MSK disease is one that has been extensively explored in the literature [8-13]. Upon review, the current literature suggests that there is a correlation between diabetes and MSK disease, especially osteoarthritis. In addition, there is increasing evidence suggesting a pathophysiological relationship between diabetes and MSK complaints, such as diabetic foot [14].

Specifically, this study will be looking at Aboriginal populations in Australia; these populations have two characteristics that make them unique to study. Firstly, they are highly understudied, and there is limited literature looking specifically at how they are affected by diabetes [15]. Furthermore, the literature would suggest that Aboriginal patients are at higher risk of diabetes and MSK disease. Consequently, this study aspires to be one of the first to analyse Aboriginal populations in this manner. 

Aims

The primary aim was to determine the relationship between diabetes severity, control and MSK disease frequency and severity. The secondary aim was to assess pain levels experienced in these patients. Then, correlating this with diabetic control and MSK complaints. Smoking status was also recorded to assess the potential impact on pain/ MSK complaints.

Background

Diabetes is the most common endocrine disorder across the globe, characterised by malfunctions in insulin response. Depending on the nature of the cause of this malfunction, diabetes is divided into two types [16]. This classification can be made using many different parameters, which are ever-changing in the literature. However, in the essence of simplicity, it can be stated that type 1 is due to an absolute lack of insulin, due to the patient being unable to synthesise the hormone. Type 2 diabetes is due to a relative lack of insulin, wherein the body is capable of synthesising bioactive hormone, but unable to respond to it, which one can call a relative lack of insulin [17]. Generally, type 1 diabetes is managed using insulin and type 2 is managed using conservative approaches and oral hypoglycaemics, most commonly metformin [18]. In severe cases, type 2 may require insulin as well. Furthermore, the diabetes was classified in severity based on NICE guidelines on HbA1c levels. Insulin-dependent type 2 diabetes is more severe than that with a HbA1c level greater than 53mmol/mol who is not taking insulin (the threshold for severe).

MSK pathology is a term that describes disorders of the bones, tendons, joints and associated tissues; these comprise a wide array of diseases. When considering MSK disease in the context of diabetes, it is vital to understand how the two can contribute to each other both in the physiological context and in terms of quality of life [19]. It is simpler to understand how diabetes can increase the intensity of MSK disease or even how diabetes may bring about de novo MSK pathology.

Looking at joint disorders, we can see that chronic hyperglycaemia ‘changes the structure and function of various extracellular proteins through increased numbers of advanced glycosylation end products (AGEs)’ [20]. These can also contribute to cytokine-mediated changes and an immune response. The exact pathogenesis is unknown. Muscular and skeletal disorders are less well understood. Despite this, one example of a well-understood pathogenesis is in the case of Charcot foot [21]. This develops because of peripheral neuropathy, leading to increased stress fractures and other deformities, which can have serious consequences on the quality of life and limb if left untreated. To summarise, MSK pathology can be exacerbated and even caused by diabetes, and in many instances, more attentive glycaemic control can greatly impact the MSK complaint(s) caused or experienced by the diabetes of a patient.

Aboriginals are one of two peoples indigenous to the Australian region. In the modern day, they are an ethnic minority and considered marginalised by some. In the medical context, they have reduced rates of primary care attendance and other disparities compared to their non-Aboriginal counterparts. With an earlier age of onset of diabetes (13.5 years on average vs. 14.8 years), as well as increased incidence, Aboriginal patients are nearly three times as likely to develop diabetes. To help combat the difference in healthcare engagement, incentives have been offered to Aboriginal peoples, like the CTG (closing the gap), as well as transport and nutritional support offered to increase primary care attendance [21-24].

Undoubtedly, the discrepancy between Aboriginal patients and non-Aboriginal patients contributes to the increased severity of the diabetes experienced by the population, as the literature suggests. However, there is also a genetic propensity in the Aboriginal population to develop type 2 diabetes. The exact cause is unknown; speculation in the literature suggests a hypothesis called the thrifty genotype. This theory posits that ancestral dietary habits lead to an increased type 2 diabetes risk. This hypothesis has been used to explain increased diabetes prevalence in other indigenous populations such as those native to the North Americas [25]. To summarise, Aboriginals are at higher risk of diabetes due to a combination of modifiable and non-modifiable factors and are under-studied, making them a prime group to observe.

## Materials and methods

This retrospective cross-sectional study collected data from 222 total patients at the Armajun trust across eight different sites. This sample size was reached by reviewing patients who had visited the trust in the last six months, of which 167 met the inclusion and exclusion criteria, an inclusion rate of 75.2%. All these patients had a confirmed diagnosis of type 2 diabetes and at least one HbA1c recording in the last year. 

The inclusion criteria included patients who had a confirmed diagnosis of type 2 diabetes at any Armajun trust primary care centre who attended the practice within the last year. The exclusion criteria are patients without accessible HbA1c readings and patients with otherwise insufficient records.

Data were collected using the Communicare patient information system about the following parameters: age, HbA1c recordings range, most recent HbA1c reading, modified pain score (see Table 1), medications, number of MSK complaints, and specific pathology (confirmed through imaging/ referral letters where possible), smoking status, and insulin usage. All data were accessed using the Armajun trust secure network at the different sites. Specifically, the software was able to generate a list of patients with a given diagnosis who had attended in the last three months, in this instance, diabetes. This approach does expose itself to recency bias, as patients who attend are more likely to be in the sample. There is potential for such patients to be more compliant with medication, which may skew the results towards the sample having better diabetic control than is representative of the whole population. Information accessed and presented was approved by the diabetes lead and a local supervisor. 

**Table 1:** Modified pain score system

Criteria	Pain score
WHO pain ladder	+1: non-opioid analgaesia, +2: weak opioid analgaesia, +3: strong opioid analgaesia, +4: multiple strong opioids
GABA agonist usage	+1: per drug
Peripheral neuropathy	+1

Secondary information regarding the degree of pain experienced by the patient was converted into the modified pain score. This approach draws from and adapts known analgesic models such as the WHO pain ladder [26] and includes specific refinements for the type of pain diabetic patients commonly experience, such as neuropathic pain [27]. That is, adding one point each for peripheral neuropathy and GABA agonist usage, as these do not fall neatly within the WHO pain ladder. GABA agonists, for example, are non-opioid but strong medications commonly used to manage chronic pain [28].

## Results

The baseline characteristics of the sample (n = 167) are shown below (Table 2). This shows all the data available for the included patients. Patients lacking information regarding smoking status were not used in the analysis for the impact this had on MSK complaints. However, provided they had sufficient HbA1c readings, they were not excluded, as smoking status assessment was a secondary aim. These data show that most patients had HbA1c levels that were above the threshold for poor control (53 mmol/mol), and on average, patients had at least one MSK complaint, with a great spread across the patients. Regarding pain, the results suggest that many patients were not experiencing pain requiring regular analgesia. For smoking status, 166 patients had data recorded; the majority were current smokers, with 111 patients having smoked before.

**Table 2:** Demographic information

Demographic	Sample: expressed as mean (SD); n = number of participants with data
Age	53.3 (13.9), n = 167
Most recent HbA1c (mmol/mol)	60.3 (24.4), n = 167
Pain score	0.45 (0.75), n = 167
Number of MSK complaints	0.90 (1.21), n = 167
Smoking status – current*	n = 67
Smoking status – ex-smoker	n = 46
Smoking status – non-smoker	n = 54

Regarding the data on the primary aim, the data were assessed based on certain HbA1c thresholds. These are adapted from local and NICE guidelines, with thresholds at <42 mmol/mol, 42-53 mmol/mol, and >53 mmol/mol for good control, adequate control, and poor control, respectively. This is detailed below, including patient numbers (see Table 3). The results suggest that patients with HbA1c >53 mmol/mol had the least number of MSK complaints, averaging just under one per patient. Patients who had moderate control averaged a similar amount, and those with good glycaemic control averaged around the same amount as the other two groups.

**Table 3:** Glycaemic control level and musculoskeletal (MSK) complaints *HbA1c categorisation is nonlinear and thus has no SD.

Criteria	Sample expressed as mean (SD), n = number of patients
HbA1c <42 mmol/mol	n = 26*
HbA1c 42-53 mmol/mol	n = 54
HbA1c >53 mmol/mol	n = 87
Insulin dependent	n = 22
MSK complaints per patient HbA1c <42 mmol/mol	1.04 (1.22)
MSK complaints per patient HbA1c 42-53 mmol/mol	1.2 (1.43)
MSK complaints per patient HbA1c >53 mmol/mol	0.64 (0.98)

There are a plethora of ways to classify MSK disease. In the interest of brevity and clarity, MSK diseases have been named by their pathology and divided into acute and chronic.

As the HbA1c value rises (worsening control), the average number of MSK complaints decreases. Patients with adequate control had the most MSK complaints, 15% more than their well-controlled counterparts. Interestingly, the most common number of MSK complaints experienced is 0 (n = 87), across all levels of glycaemic control. Observing patients who had at least one MSK complaint, the ranking of means remains the same. The number of MSK complaints per patient changes to 1.65, 2.28, and 1.56 for patients with good, adequate, and poor control, respectively. The poor control group had the highest ratio of patients without any MSK pathology (1), followed by the adequate (0.98) and then well-controlled (0.35). 

The three most common chronic MSK pathologies experienced were osteoarthritis, chronic lower back pain, and gout, with 31, 20, and 17 patients, respectively. Chronic conditions were much more common (see Tables 4-5), with the most common acute conditions being equally interspersed between all levels of glycaemic control. The most common acute conditions were herniated discs, rotator cuff pathology, ankle fractures, and fractures in the hand and wrist, with five, four, three, and three cases, respectively (see Table 5). 

**Table 4:** List of frequency of chronic musculoskeletal (MSK) complaints

MSK complaint	Frequency
Osteoarthritis	31
Chronic lower back pain	20
Gout	17
Carpal tunnel syndrome	5
Trochanteric bursitis	5
Plantar fasciitis	4
Osteopaenia	4
Sciatica	4
Subacromial bursitis	4
Fibromyalgia	2
Restless leg syndrome	2
Rheumatoid arthritis	2
Diabetic foot	1
Tenosynovitis	1
Adhesive capsulitis	1
Perthe’s disease	1
Charcot foot	1
Trigger finger	1

**Table 5:** List of frequency of acute musculoskeletal (MSK) complaints

MSK complaint	Frequency
Disc herniation	5
Rotator cuff pathology	4
Fracture (foot and ankle)	3
Fracture (hand and wrist)	3
Meniscal injury	2
Fracture (neck of femur)	2

Pain varied across different levels of glycaemic control (see Table 6). Patients who had more MSK complaints had higher scores. For example, in patients with good control, those without MSK pathology had an average pain score of 0. Those with MSK pathology averaged a pain score of 1.11. In patients with adequate control, the average pain score increases from 0.44 to 2.28 when patients without MSK complaints are removed from the sample. Similarly, in patients with poor diabetic control, the average pain score increases from 0.35 to 0.91 when patients without MSK disease are removed from the sample.

**Table 6:** Pain scores

Criteria	Sample expressed as mean (SD), n = number of patients
Pain score in <42 mmol/mol	0.70 (1.20)
Pain score in 42-53 mmol/mol	0.43 (0.63)
Pain score >53 mmol/mol	0.35 (0.63)
Pain score = 0	n = 115
Pain score = 1	n = 42
Pain score =2	n = 9
Pain score = 3	n = 2
Pain score = 4	n = 2

The patient cohort with poor control had the highest percentage of patients with a pain score of 0, with 72.4% of patients having a pain score of 0 (n = 63). The next highest was the patients with good control, 65.3% a pain score of 0 (n = 17). In patients with adequate control, 61.4% had a pain score of 0 (n = 35). The maximum pain score of any patient was 4, with good glycaemic control and a history positive for two joint replacements and osteoarthritis. As for those who scored a three on their pain score, one had good glycaemic control, and the other had poor glycaemic control. The first had osteoarthritis and chronic lower back pain and was a smoker with the most recent HbA1c at 32.2mmol/mol. However, the highest recorded was 63 mmol/mol, well within range for poor control. As for the latter, this patient had a history of disc degeneration and osteoarthritis. Their most recent reading was 55.2 mmol/mol, with their highest being 57 mmol/mol.

Insulin-dependent type 2 diabetes is the most severe, representing tertiary management in diabetics NICE guidelines [17]. From the sample (n = 167), 22 patients were insulin dependent, one patient had adequate control, and the remainder had poor control per their most recent HbA1c reading. The average number of MSK complaints among insulin-dependent diabetics was 0.45 (0.80) (expressed as mean (SD)), with 15 patients experiencing 0 MSK pathology. The average pain score in these patients was 0.23 (0.53). Among patients who had MSK complaints, the average number of complaints was 1.43. The nature of the complaints varied greatly with acute and chronic conditions, with no conditions being shared among any insulin-dependent patients. Pain in insulin-dependent patients was similar; in patients with MSK pathology, the average pain score was 0.71.

## Discussion

MSK complaints in diabetes contribute to significant impacts on patient quality of life and are one of the most common comorbidities of diabetes. Details and protocols on Australian Aboriginal diabetic care remain sparse in the current literature. Consequently, this study represents new insight into the effects glycaemic control has specifically on MSK complaints and pain experienced by Australian Aboriginal diabetic patients. In comparison to the existing literature, this study observes fewer patients, but the average demographics of the patients match in terms of age, smoking status, past history, and other relevant metrics [20,25-26]. The results from this study show that the degree of glycaemic control in Aboriginal patients does not necessarily correlate with the number of MSK complaints experienced, whether this be acute, chronic, or any otherwise classification. As is evident in the average number of MSK complaints in each of the three levels of glycaemic control. Patients who had the poorest glycaemic control (n = 83) averaged the lowest number of MSK complaints (0.60). Moreover, this group also had the smallest standard deviation, meaning that relatively more patients had fewer MSK complaints. Taking this one step further, if one analyses insulin-dependent diabetics (n = 22), this group had the lowest average number of MSK complaints of the whole sample (0.45), and the lowest standard deviation at 0.80. However, the small number of patients in this group does limit the power of this conclusion.

Moreover, this sample suggests that there is no specific MSK complaint that is more likely to be caused by poorer glycaemic control, as the MSK complaint type and frequency mirror those of well-controlled diabetic and non-diabetic populations. What is more interesting is how the findings contrast with the established literature. At least in non-Aboriginal populations, it appears that glycaemic control is well correlated with MSK disease incidence and severity. So much so, in fact, that in the established guidance for diabetics with any additional pathology, MSK or otherwise, oftentimes among the first steps of management is to optimise diabetic control using the appropriate medications [29]. The cause behind this disparity could be due to several reasons, one of which is the inherent difference in Aboriginals, both in terms of genetics and their access to healthcare. Other possible causes could, of course, be due to weaknesses in the study design, including a smaller sample size, especially when compared to other literature on the same topic [20,27-30].

The secondary aim tells a similar story. Firstly, looking at pain levels, average pain levels seem to either remain similar or decrease with worsening glycaemic control. Evidenced by the highest average pain score (0.70) being in the group with the best glycaemic control, the group with the worst glycaemic control having both the lowest average pain score, and the highest percentage of patients with a pain score of 0. However, in the group with good glycaemic control, the standard deviation is almost double that of the other groups (1.20 vs. 0.63 and 0.63). Narrowing down the cause, we observe that the three highest pain score patients are in this group. Among them is a patient with a history of multiple joint replacements and another with a history of clavicular fracture. As the total number of patients with good control is the smallest, even if these two patients can have a great impact on the average pain score. If these patients are removed, then the average falls to 0.46, which is much closer to scores found in other groups, although still the highest. Whether or not it is appropriate to exclude these patients is another question entirely.

One of the strengths of this study is the level of detail in which patients were analysed and data collected. Firstly, all data were collected across one trust, limiting differences and errors in data recording and data access. Furthermore, as the trust contains many Aboriginal patients, the number of attendees in the last year (which formed the initial sample (n = 222)) is great; hence, the sample size was as large as feasible. Patients were analysed both on an individual basis when necessary and as stratified groups, in terms of glycaemic control level. Another strength was the recording of HbA1c ranges of patients, which proved useful when analysing potential anomalies.

The main weakness of the study is the inherent bias when selecting patients who have been seen in the last year. Such selection bias was difficult to avoid, as the preliminary list of patients accessed through the Communicare patient software was only available across sites using this method. Attempting to generate a list of all diabetic patients at the trust was not feasible. Moreover, there is likely to be human error in confirming the diagnoses, as some were supported with imaging, and others were not. It is also unclear how long different patients have been members of the practice, and how up-to-date their data are. There were also methodological issues, like in the modified pain score system. Patients experiencing pain may not take analgesia to this effect, let alone whether this was sufficient to properly manage said pain. Stratifying the patients based on their most recent HbA1c reading also poses problems, as this reading may have been an outlier. However, there was no other appropriate way to classify the severity that was not already used, the ranges were not associated with dates, and some patients had no HbA1c ranges on their records altogether. 

Expanding further on this, despite the sub-optimal method of data collection, it is difficult to draw conclusions that hold any significant weight. Primarily, this is due to the extremely small sample size of patients, as well as the bias in collecting this number, as mentioned above. The above explains why the results of this study contrast so heavily with what we know from the existing literature, which is that improving glycaemic control improves MSK complaint incidence and severity. 

The reason behind this difference can be due to one of two main reasons: either that Aboriginal populations have inherent genetic/epigenetic differences, such that they exhibit the opposite trend, or, more likely, that the weaknesses of this study mean that the conclusion drawn is not representative of the population. Ultimately, further research into Aboriginal groups and their relationship with diabetes is required. However, this study is able to impart a potential baseline and framework for attempting analysis into such groups and how their unique characteristics shape their incidence and severity of MSK complaints, in light of how type 2 diabetes mellitus impacts them. 

## Conclusions

This study finds that MSK complaints in diabetic Aboriginals are not correlated to the degree of glycaemic control the patient exerts, in terms of quantity or quality of problem(s) experienced. As far as pain is concerned, similar deductions can be made. Patients with poorer glycaemic control did not experience more pain than those with better glycaemic control. The intriguing nature of these postulations raises questions. Most notably, what is the true cause behind the discrepancy in this sample, especially compared to non-Aboriginal populations? It is entirely possible that much these inconsistencies can be explained by study design and execution imperfections, including its small size. However, it is not possible to know this for certain, at least when viewing this study in isolation. What this study contributes to the literature could be viewed as an introduction to diabetic aboriginals and their expression of MSK ailments, as one of the few to do so. Moreover, the possibility of Aboriginals expressing/ experiencing diabetes differently due to an inherent difference, resulting in the changes seen, poses a challenging question to the existing discussion surrounding diabetes. Further research questioning into how glycaemic control impacts the co-morbid status of Aboriginal populations could assist in answering these questions. MSK complaints were the topic of study in this area, but analysing other comorbidities may help uncover additional insights into whether Aboriginal populations have a different response to diabetes and the pathophysiological impact it exerts upon the body. Ultimately, more additions to the literature will serve well in answering the questions this study has raised.
